# Intergroup alliance orientation among intermediate-status group members: The role of stability of social stratification

**DOI:** 10.1371/journal.pone.0235931

**Published:** 2020-07-24

**Authors:** Luca Caricati, Gianluigi Moscato, Chiara Bonetti

**Affiliations:** 1 University of Parma, Parma, Italy; 2 University of Malaga, Málaga, Spain; Fordham University, UNITED STATES

## Abstract

Three studies have tested the hypothesis that intermediate-status groups are more oriented to ally with outgroups when their social position is under threat. In study 1, participants believed that their ingroup was intermediate in status and social stratification was manipulated as either stable or status-detrimental unstable. Results indicated that participants were more likely to seek alliances a) with a high-status group and b) when social stratification was status-detrimental unstable. Study 2 showed that participants were more likely to seek alliances with a lower status group when social stratification was status-detrimental unstable rather than stable, while they were supportive of policies helping disadvantaged groups regardless of the stability of social stratification. Study 3 showed that when social stratification was status-detrimental unstable, intermediate-status group members were more oriented to ally with a low-status group, equally supportive of policies helping disadvantaged groups, but less oriented to supplying direct help to a low-status group.

“*Closure is not inherited; it is achieved through strategies of social effort that individuals use as they attempt to reproduce their social-class position*”

[1]

## Introduction

The triadic social stratification theory [2,3] focalizes on intermediate-status groups, that is, groups that, in comparison with at least two other groups, occupy the social position that is in-between. TSST is rooted in the social identity theory [4] and assumes that intermediate-status groups can provide positive social identity because members can compensate the negative upward intergroup comparison with the positive downward intergroup comparison [e.g., 5,6]. Thus, intermediate-status group members are expected to be motivated to maintain the existing social stratification, given that they can obtain positive social comparison with lower-status groups. A central tenet of TSST is that the stability of social stratification plays an important role for the intergroup behavior of intermediate-status group members. Given that social identity is satisfied through downward comparison, intermediate-status group members are expected to have no, or little, motivation to compete with other groups when social stratification is stable. When social stratification becomes unstable, intermediate-status group might instead experience a threat to their social identity, and this should be especially evident when instability concerns differences with low-status groups (i.e., status-detrimental instability). In such a case, TSST expects that intermediate-status group members might act in a reactionary way, trying to defend and maintain their intermediate position although it is not completely advantageous. More precisely, one of the hypotheses of TSST is that the intermediate-status group would be primarily concerned with preventing falling than with trying to increase its social position by challenging superior groups [2,5–8]. Accordingly, it has been shown that intermediate-status group members are more biased against both high-status and low-status outgroups when social stratification in depicted as unstable rather than stable [e.g., 7].

All the evidence hitherto discussed refer to competitive behaviors such as intergroup bias and discrimination. To the best of our knowledge, no studies have considered non-conflictual intergroup behaviors within triadic social stratification. Recently, some authors have investigated the possibility that a “third party group” may participate in collective actions, highlighting the role of alliance between minorities or group mobilization, favoring a disadvantaged *outgroup*. For example, Klavina and van Zomeren [9] showed that some groups were willing to engage in collective action, helping *another* disadvantaged outgroup to the extent that members identified with the outgroup’s cause, experienced anger, and perceived themselves to be efficacious (e.g., Latvians’ motivation to collectively protest against the Russian annexation of Ukrainian territory). Moreover, to summarize a richer pattern of results, Dixon et al. [10] showed that positive contact promoted political solidarity among disadvantaged groups of Indians toward their (disadvantaged outgroup of) Black neighbors. Similarly, Dixon et al. [11] found that intergroup contact among members of different disadvantaged groups in Indian society increased the intention of historically disadvantaged groups of Muslims to endorse collective action to resist or change shared inequalities. Interestingly, this tendency was weakened by the positive contact that Muslims had with the historically powerful group of Hindus.

In the examples thus far discussed, the third group was either an external-hierarchy group that decided to support a disadvantaged outgroup [9] or a disadvantaged minority that allied with another minority [10,12]. These works represent an important advancement for the understanding of complex dynamics occurring in intergroup relations. However, little is known about the alliance orientation among groups that possess different statuses within the same triadic social hierarchy [13]. The possibility of analyzing strategies of cooperation among groups is an advantage that derives from considering more than two groups within social stratification. As stated by Dixon et al. [11], “most societies are organized not in terms of simple majority versus minority relations (e.g., black–white, immigrant–host, gay–straight), but in terms of more complex relations, marked by a multiplicity of status distinctions and patterns of allegiance, hostility, and discrimination” (p. 85). If one takes into account a “binary” stratification (i.e., high- vs. low-status groups) or a binary path of influence and power (dominant vs. dominated), one can only expect competition or “submission and dominance” between groups [see also 13]. A triadic social stratification approach, in contrast, permits taking into account different patterns of competition and alliance between groups within the same social hierarchy [2,3]. Accordingly, the aim of this paper was to consider intergroup alliance orientation of intermediate-status group members when status differences were either stable or status-detrimental unstable. The primary research question that drove this work was: are intermediate-status group members more oriented to ally with other groups when they are assured of their social position (i.e., stable social stratification) or when they experience a threat from below (i.e., status-detrimental instability)? The answer to this question might seem easy, given that stable social stratification should reduce intergroup competition and, then, it should be the condition in which intergroup alliance orientation would be more likely to emerge. Indeed, provided that social identity can be made positive through downward comparison, it can be expected that intermediate-status group members would be more oriented to ally with higher and lower status groups when social stratification is stable and threat to social identity is reduced. This, however, might be not the whole story. Indeed, it is worth noting that, for intermediate-status groups, alliance could mean allying either with a higher status group, a lower status group, or both. For this reason, for intermediate-status groups, alliance could not be seen simply as an example of collective protest against dominant groups or an attempt to change the status quo. It can also operate as a way of maintaining the existing social stratification: through alliance with other groups, intermediate-status group members can negotiate social change and try (or hope) to maintain the possibility of comparing positively with lower status groups. This aspect may lead to a paradoxical eventuality: intermediate-status group members can be even more motivated to seek alliance with other groups when social stratification is unstable and their social position is threatened. Alliances with other groups, indeed, might be a way to reduce the threat of worsening their social position. We speculated that, for groups that are hierarchically “in-between”, and hence have some level of socio-psychological advantage, alliance with outgroups could be a way through which members hope to maintain, or not worsen, their social position. Thus, we expected that when differences with a low-status group are eroded (i.e., there is threat to social identity), intermediate-status groups would be more oriented to ally with other groups with respect to a condition in which status differences are stable. We have tested this general proposition in three studies.

## Overview of the current research

The three studies tested the effect of instability of social stratification on alliance orientation of intermediate-status group members. In Study 1, we induced participants to believe that their nation was intermediate in status and that this position was either stable or status-detrimental unstable (i.e., threatened by the low-status group). We then measured the extent to which participants agreed to an ingroup alliance either with high- and low-status outgroups. In Study 2, we replicated the research design of Study 1, but focusing attention on a low-status target and measuring alliance orientation, support for policies to help disadvantaged groups, and direct help to the low-status outgroup. The latter measures were added in an attempt to better qualify the process of alliance-seeking in intermediate-status group members. Indeed, if alliance orientation is motivated by the desire to maintain the status quo facing status-detrimental instability, then direct help to the threatening outgroup and support for policies helping the disadvantaged outgroup (but without altering the existing social hierarchy) should not increase when the threat comes from below. Finally, in Study 3, we applied the research design of Study 2 to a sample from a different nation.

## Study 1

Study 1 aimed at preliminarily testing the hypothesis that an intermediate-status group will be more likely to ally with higher and lower status groups in a status-detrimental unstable condition than when social stratification is stable.

### Materials and methods

#### Design

We used an experimental design in which status was induced to be intermediary and stability was manipulated to be either stable or status-detrimental unstable. The dependent variable was alliance orientation, with both higher and lower status groups. The research design was thus 2 (stability: Stable vs. Unstable) x 2 (target: Higher vs. Lower status group), with the latter as a within-participant factor.

#### Sample size calculation

We considered an effect size from low to medium (*η*^2^ = .02), alpha = .05 and power = .80. With these parameters and considering 2 groups and 2 measurements as well as a correlation between repeated measures of .50 (default in G*Power), the required sample size was 100, as indicated by G*Power [14].

#### Participants

We obtained answers from 155 participants. Of them, 21 did not conclude the procedure, 13 failed the status manipulation check and were therefore excluded. One further participant was excluded because he/she declared having a different citizenship. The analyzed sample is thus composed of 120 participants (62.5% women). Unfortunately, age was not recorded due to an error in the survey. With the same criteria used for computing the required sample size and considering real correlation among repeated measures (see below), sensitivity power analysis indicated that the minimum detectable effect size was *η*_*p*_^*2*^ = .024.

#### Procedure

The procedure was administered online. Participants were invited by mail to take part in a research about recall of press articles. After informed consent, participants learned that the experiment required them to read a short paper, let some time go by, and then answer some questions about the paper. They were then invited to read the paper carefully. The content of paper was used to induce the status of the ingroup and changed to manipulate the stability of social stratification. The article (see S1 Fig), which was attributed to an influential national journal, was about the economic situation of three European countries, namely Italy (intermediate-status ingroup), Germany (high-status outgroup), and Greece (low-status outgroup). The article explained that Germany was a powerful and economically strong nation in the UE, Greece was a powerless and economically weak country, whereas Italy was depicted as intermediary in power and economic force. Depending on stability condition, the paper explained that things would either remain the same (stable condition, n = 62) or they would change, and Greece was approaching Italy in terms of gross domestic product (GDP) (status-detrimental unstable condition, n = 58). The article concluded that Greece was either approaching (or distancing itself from) Italy, and this produced worry (serenity) about the future of Italy in terms of political and economic power within the UE. In order to make the story clearer, a graph depicting the trends of Italy’s and Greece’s GDP in the last year was supplied at the end of the paper. In the status-detrimental unstable condition, the trend of Greece’s GDP tended to increase and approach that of Italy. In the stability condition, the trend of Greece’s GDP tended to remain stable over time. The participants then learned that some time had to go by before performing the memory task and they were invited to answer some questions about their opinion of their nation. After the dependent variables were administered, participants answered questions supposedly measuring their memory and some demographic questions. At the end, participants were informed that the paper was fictitious and contained false news, and the real aim of the research was disclosed. In agreement with the ethical statement of the National Association of Psychology, participants were invited to supply further consent to use the data collected after being made aware of the deception that was used in the research.

#### Measures

*Alliance orientation*. Alliance orientation with other groups was measured with six items on a 7-point rating scale (1 = *completely disagree*; 7 = *completely agree*). Three items referred to alliance with the higher status group and three items referred to alliance with the lower status group (i.e., “Italy would benefit from an alliance with Greece/Germany”; see Supporting Information).

*Manipulation check*. In order to determine whether participants were aware of the position of their ingroup, we used a question in which they choose whether Italy had a “higher status than either Germany or Greece”, a “lower status than either Germany or Greece” or an “intermediary status with respect to Germany or Greece” [For a similar procedure, see 15].

Given Hauser, Ellsworth, and Gonzalez’s [16] claim against the use of manipulation checks in social psychology as the effects of manipulated variables could be confounded, we decided to test effectiveness of instability manipulation by using a related construct of status instability, namely perception of threat (assuming that threat should be stronger in the status-detrimental unstable condition). Instability manipulation was then checked with 4 items on a 7-point rating scale (1 = *completely disagree*; 7 = *completely agree*), asking participants to express their agreement with a statement such as “What is described in the article is a threat to my nation” and “I feel that what is described in the article is an attack on my nation”. Reliability was not good (α = .59). For each scale, the items were presented in random order.

### Results

#### Preliminary analysis

Firstly, we analyzed the psychometric properties of the scale of alliance orientation with exploratory factor analysis with principal axis factoring and promax rotation. Unexpectedly, the analysis yielded three factors with eigenvalue greater than 1, and inspection of loadings revealed that the third factor was composed of the two reverse-score items (see S1 Table). Accordingly, the reliability of the three-item scale of alliance with the low-status group was low (α = .50), whereas the reliability was acceptable for alliance with the high-status group (α = .61). We therefore decided to exclude the reversed items. Thus, factor analysis revealed the existence of two factors, explaining 77% of the variance, with improved reliability for alliance with both the lower (α = .63, *r* = .46, *p* < .001) and the higher (α = .77, *r* = .62, *p* < .001) status groups. Measures of alliance orientation were weakly correlated with each other (*r*(120) = .27, *p* = .003).

#### Manipulation check

Analysis of variance (ANOVA) revealed that threat was stronger in the status-detrimental unstable condition (*M* = 2.84, *SD* = 1.01) than in the stable condition (*M* = 2.23, *SD* = 0.97), *F*(1, 118) = 11.53, *p* = .001, *η*_*p*_^*2*^ = .09. Thus, it appeared that the manipulation of stability created different levels of perceived threat and seemed to be effective.

#### Stability and alliance orientation

A 2 (Stability) x 2 (Target) mixed ANOVA was performed in which scores of alliance orientation were introduced as a dependent measure. Results (see Table 1) indicated that alliance orientation with the higher status group (*M* = 4.03, *SD* = 1.45) was stronger than alliance orientation with the lower status group (*M* = 3.44, *SD* = 1.30), *F*(1, 118) = 14.87, *p* < .001, *η*_*p*_^*2*^ = .12, and this effect was independent of the condition of stability, *F*(1, 118) = 0.24, *p* = .876, *η*_*p*_^*2*^ < .01). Moreover, a main effect of stability emerged, *F*(1, 118) = 4.92, *p* = .028, *η*_*p*_^*2*^ = .04, for which, as expected, alliance orientation (with both outgroups) was stronger in the status-detrimental unstable condition (*M* = 3.96, *SD* = 1.15) than in the stable condition (*M* = 3.52, *SD* = 1.01). Note that similar results were obtained considering the three-item version of the alliance orientation scales (S2 Table).

**Table 1:** Means and standard deviations of alliance orientation according to target outgroup and stability condition.

	Alliance orientation					
	High-status group		Low-status group		Total	
	*M*	*SD*	*M*	*SD*	*M*	*SD*
**Status-detrimental Unstable**	4.27	1.49	3.66	1.24	3.96	1.15
**Stable**	3.81	1.39	3.24	1.33	3.52	1.01
**Total**	4.03	1.45	3.44	1.30	3.74	1.10

#### Supplementary analysis

In order to explore whether the effect of instability manipulation on alliance orientation could be due to a perceived threat to social position, we performed a mediation analysis in which the perceived threat mediated the relationship between instability and alliance orientation (with both outgroups together). We adopted the framework recommended by Yzerbyt, Muller, Batailler and Judd [17] using JSmediation package in R. Results indicated that the perceived threat mediated the relationship between instability and alliance orientation (point estimate: 0.12, 95%CI[0.004; 0.29]), turning the effect of instability (stability condition coded as 0) to not significant, *b* = 0.31, *SE* = 0.20, *t*(117) = 1.54, *p* = .127.

### Discussion

Study 1 supplies the first evidence that intermediate-status group members are more likely to seek an alliance with other groups when they perceive a threat to their social position. Results indicated that alliance orientation toward a higher status group was stronger than alliance orientation toward a lower status group. This pattern was independent of the stability of social stratification, but stability affected the extent to which intermediate-status group members were oriented to ally with the two outgroups. More precisely, when social stratification was status-detrimental unstable, intermediate-status group members were more oriented to ally with both outgroups than when social stratification was stable. There was a suggestion that this effect was also mediated by a perceived threat. These evidences support the idea that perceived threat motivates intermediate-status group members to try to maintain their social position (i.e., to avoid “falling”). On the whole, results from Study 1 seem to be congruent with the TSST view that intermediate-status groups are motivated to defend and maintain the existing social stratification and more likely to support dominant groups than to support dominated groups [2,7].

Study 1 has some important shortcomings that should be acknowledged. First, the measure of alliance orientation had some psychometric weakness, as the scales were not very reliable. Similarly, some items about manipulation check (i.e., perceived threat) were not very reliable. We tried to correct these shortcomings in Study 2.

## Study 2

Study 2 was carried out in order to replicate and extend the results from Study 1. In our opinion, the most interesting (and perhaps paradoxical) evidence from Study 1 is the increased intermediate-status group members’ alliance orientation with low-status (and threatening) outgroup when status differences were status-detrimental unstable. Accordingly, in Study 2 we focused on intermediate-status group members’ alliance orientation with a low-status group only, and tried to asses in more detail the hypothesis that alliance orientation is motivated by the desire to maintain social position. This choice was based on several reasons. First, being an attempt to investigate new and never-analyzed processes, we were aware that we had to proceed in steps to collect evidence. Hence, in order to accumulate solid scientific evidence, we decided to focus our (limited) resources and attention on the relationship between intermediate- and low-status groups. This leads to the second reason, that is that the alliance between disadvantaged groups (i.e., intermediate- and low-status groups) is more interesting given that it can be considered as having the potential to drive social change attempts. Moreover, an alliance with a low-status group appears to require more investigation than an alliance with a high-status group. Indeed, the fact that intermediate-status group members, when threatened from below, may seek an alliance with threatening disadvantaged groups might be interpreted either as an attempt to join forces to change social stratification or as an attempt to prevent the possibility to worsen social position. The attempt to elucidate these two alternatives was at the core of our reasoning. Indeed, following TSST, we speculated that if intermediate-status group members seek alliances with a low-status group in order to maintain the status quo and defend their relatively advantaged social position, then they should be more oriented to ally with a low-status group when the low-status group represents a threat than when social stratification is stable. This may occur because, by means of alliance-seeking, intermediate-status group members might believe they create dependency in the low-status group [18,19] and thereby prevent the risk of further worsening their social condition. We also speculated that, if intermediate-status group members support alliances with a threatening low-status group because they hope to maintain their social position, then alliances should take a specific form in which direct help to threatening low-status groups is limited or avoided. In other words, we reasoned that if alliance is strategically aimed at maintaining a status differential between intermediate- and low-status groups, then intermediate-status group members would avoid directly helping low-status groups to improve their condition given that this would not reduce (and even increase) the threat. If the alliance were motivated to join forces with a low-status group, then one would expect the alliance orientation with the low-status group to be accompanied by an increasing willingness to directly help the low-status group to improve. In Study 2 we added items measuring participants’ support for direct help to a low-status group in order to observe whether any changes occurred when social stratification was depicted as stable or status-detrimental unstable. Finally, we also speculated that effect of stability of social stratification should be specific for dimensions that are related to the relative position of the intermediate- and low-status groups (e.g., alliance and direct help). In other words, we speculated that the effect of stability/instability should not appear when intermediate-status group members evaluate the possibility that *both* intermediate- and low-status groups would improve their condition given that social stratification remains the same (i.e., both disadvantaged groups could improve, but their relative position remains the same). In order to capture this aspect, in Study 2, using the same cover story as in Study 1 (i.e., a description of the trend of national relations among three European nations), we added a measure of support for EU policies aimed at favoring “less rich” European countries. Our main expectation was that agreement for supporting policies would be not (or would be little) affected by the stability of social stratification (given that such policies, which help intermediate-status groups and less rich groups, should not affect status differences between intermediate- and low-status groups).

To sum up, if intermediate-status group members are motivated to preserve the status quo, we expected that alliance orientation with a low-status group should be stronger when social stratification is status-detrimental unstable, but support for helping policies and direct help for the threatening outgroup should not. In statistical terms, a significant interaction between stability of social stratification and alliance versus direct help versus support for helping politics was expected.

In Study 2, we also tried to correct some shortcomings of Study 1 and to develop a set of items that would measure alliance orientation more reliably. Similarly, given problems in Study 1, we tried to check the manipulation of stability directly in order to be more certain of its effectiveness.

### Materials and methods

#### Design

The design was similar to that used in Study 1 except for the dependent variables, which were the orientation to ally with the low-status group only, direct help for the low-status group, and support for helping policies. The research design was thus a 2 (stability: Stable vs. status-detrimental unstable) x 3 (Alliance orientation vs. direct help vs. support for helping policies) with the last one as within-participant factor.

#### Sample size calculation

Given the differences in the dependent measures, results from Study 1 were not useful for determining sample size. To be conservative, we halved the effect size and considered a low effect size (*η*_*p*_^*2*^ = .01), α = .05, and power = .80. With these parameters and considering 2 groups and 3 measurements and a correlation between repeated measures of .30, the required sample size was 226.

#### Procedure and participants

The procedure was similar to that used in Study 1 except that participants were university students from several courses at a public university. Participants were randomly assigned to either status-detrimental unstable (*n* = 140) or stable (*n* = 126) conditions.

There were 747 contacts to the research page but only 471 participants decided to start the research. Of them, 176 did not conclude the procedure, and 13 failed the status manipulation check and were therefore excluded. Ten further participants were excluded because they declared they had a different citizenship, and six further participants did not supply the final consent to use their answers. The analyzed sample is thus composed of 266 participants (84.2% female, mean age = 26.98, *SD* = 9.34, range = 19–68). With the same criteria set for previous power analysis and considering the real correlation between measurements (see below), sensitivity power analysis indicated that the minimum detectable effect size was *η*_p_^*2*^ = .01.

#### Measures

*Alliance with the lower status group orientation*. This was measured with three items on a 7-point rating scale (1 = *completely disagree*; 7 = *completely agree*). Example of items is “I am in favor of an economic and political alliance between Italy and Greece” (see Supporting Information for all items).

*Helping the outgroup*. This was measured with two items on a 7-point rating scale (1 = *completely disagree*; 7 = *completely agree*) which asked about direct help or support from Italy toward Greece: “If Greece asked for greater economic aid from Europe, Italy should support Greece” and “I am in favor of a policy to help Greece’s economy to grow”.

*Support of helping policies*. This was measured with four items on a 7-point rating scale (1 = *completely disagree*; 7 = *completely agree*). Items were “I am in favor of a policy of redistributing the national debt in favor of the less wealthy European nations”, “If Europe would decide to economically help the less wealthy European countries, I would agree”, “I am in favor of a policy that helps the economy of less wealthy European countries to grow”, and “The European Union should help the richest nations maintain and increase their well-being rather than helping the less wealthy nations” (reversed).

*Interests of ingroup and outgroup*. One item on a 6-point rating scale asked participants to indicate the extent to which they believed that the interest of Italy and Greece were very different (1) or very similar (6). This measure was added as control variable in order to observe whether alliance orientation and support for helping policies were correlated with perceived similarity of ingroup and outgroup interests.

*Allophilia*. We also administered eight items taken from the affect (e.g., “In general, I have positive attitudes about Greece”) and engagement (e.g., “I am truly interested in understanding the points of view of Greece citizens”) dimensions of the Allophilia Scale [20]. Reliability was good (α = .91). This scale was administered in order to control for the effect of general positive feeling towards the outgroup.

*Manipulation check*. In order to check whether participants were aware of the position of their ingroup, we used the same check item as in Study 1. Instability manipulation was checked with two measures. The first one, as in Study 1, used 4 items rated on a 7-point scale (1 = *completely disagree*; 7 = *completely agree*) asking participants to express their agreement with the following statements (which were partially reworded following Study 1 results): “The situation described in the article represents a threat to Italy”, “The situation described in the article is worrying”, “I have the impression that Italy is devalued by the situation described in the article”, and “I think the Italian citizens would be worried if they read the information in the article”. Reliability was fairly good (α = .71). A further item on a 5-point rating scale asked participants to indicate the extent to which they believed that differences between Italy and Greece were worse for Germany (1), the same (3), or better for Italy (5) in the future.

For another research project and for exploratory purposes, in studies 2 and 3, we also administered a scale of system justification, social dominance orientation, national identification and right-wing orientation.

### Results

#### Preliminary analysis

Firstly, we analyzed psychometric properties of the scales of alliance orientation, direct help, and support for helping policies with exploratory factor analysis with principal axis factoring and promax rotation. Unfortunately, the analysis did not support the expected three dimensions. Inspection of factor loadings revealed that the items measuring direct help were not arranged on an independent and single dimension, but loaded on two other factors (see S3 Table). Accordingly, confirmatory factor analysis (maximum likelihood with robust standard error estimation) was unsupportive for the three-dimension structure, *χ*^*2*^(24) = 114.54, *p* < .001, CFI = .84, TLI = .77, RMSEA = .12, *p* < .001, 95% CI [.10, .14]. Thus, we decided to exclude these two items and not consider the measure of direct help and limit our analysis to alliance orientation and support for helping policies. Confirmatory factor analysis of the remaining items supported the expected two-factor structure, *χ*^*2*^(13) = 37.03, *p* < .001, CFI = .95, TLI = .92, RMSEA = .08, *p* = .030, 95% CI [.06, .11], and indicated that all the items were adequately measured by the intended latent trait (all *p*s < .001). Accordingly, reliability was good for alliance orientation (α = .84) and acceptable for helping policies (α = .65). We then computed the scores of alliance orientation and support for helping policies as latent trait scores. The two scores correlated with each other (*r* = .32, *p* < .001). Importantly, alliance orientation correlated positively with similarity of interest of Italy and Greece (*r* = .27, *p* < .001), whereas support for helping policies did not (*r* = .03, *p* = .63). The differences between these two correlations was hardly imputable to hazard (*Z* = 2.83, *p* < .001). This seems to indicate that alliance orientation and support for helping policies are two distinct constructs, and that alliance orientation is linked to the interests of the ingroup. Table 2 depicts the descriptive statistics and zero-order correlations for the considered measures.

**Table 2:** Descriptive statistics and zero-order correlations for the considered measures (Study 2).

	*M*	*SD*	2	3	4
**1 Alliance orientation**	.00	1.17	.32**	.27**	.20**
**2 Support for helping policies**	.00	0.91	-	.03	.21**
**3 Similarity of interests**	3.59	1.05		-	.16*
**4 Allophilia**	5.20	1.14			-

*N* = 266.

* *p* < .05.

** *p* < .01.

Experimental conditions were equivalent for gender, *χ*^*2*^(1) = 0.04, *p* = .838, mean age, *F*(1, 264) = 0.01, *p* = .901, and Allophilia, *F*(1, 264) = 0.28, *p* = .596.

#### Manipulation check

Replicating finding of Study 1, ANOVA revealed that threat was stronger in the status-detrimental unstable condition (*M* = 4.61, *SD* = 0.95) than in the stable condition (*M* = 2.94, *SD* = 1.21), *F*(1, 264) = 157.72, *p* < .001, *η*^*2*^ = .37. ANOVA also indicated that participants in the status-detrimental condition were more likely to believe that the condition of the ingroup would worsen in the future (*M* = 2.41, *SD* = 1.07) than participants in the stable condition (*M* = 3.65, *SD* = 1.04), *F*(1, 264) = 90.86, *p* < .001, *η*^*2*^ = .26. Thus, manipulation of stability appeared to be effective.

#### Hypothesis testing

**Fig 1:**
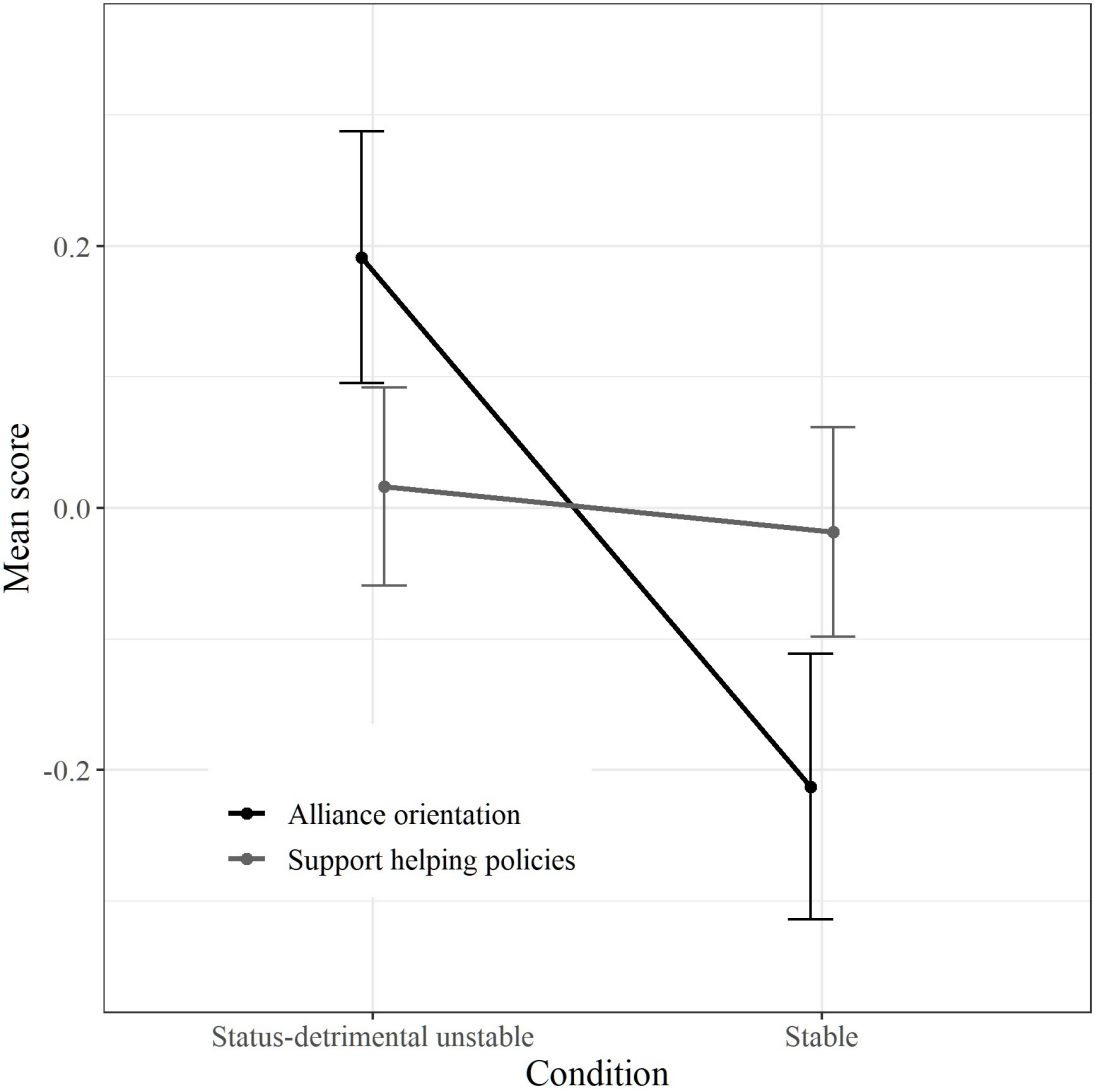
Mean scores of alliance orientation and support for helping policies according to stability conditions (Study 2). Bars represent standard errors.

A 2 (Stability) x 2 (Alliance vs. Support for policies) mixed ANOVA was performed with allophilia as covariate (analysis without covariates provided the same results; see S4 Table). Results indicated an effect of stability, *F*(1, 263) = 4.76, *p* = .030, *η*^*2*^ = .02, for which scores were generally higher in the status-detrimental unstable condition (*M* = 0.10, *SE* = 0.07) than in the stable condition (*M* = -0.12, *SE* = 0.07). This effect was due to alliance orientation as indicated by the Stability x Alliance-support for policies interaction, *F*(1, 263) = 5.99, *p* = .015, *η*^*2*^ = .02 (see Fig 1). More precisely, and in line with expectations, whereas support for helping policies was similar in stable (*M* = -.02, *SE* = 0.08) and status-detrimental unstable conditions (*M* = .02, *SE* = 0.08) *F*(1, 263) = 0.10, *p* = .753, *η*^*2*^ < .01, alliance orientation was higher when social stratification was status-detrimental unstable (*M* = 0.19, *SE* = 0.10) than when it was stable (*M* = -0.21, *SE* = 0.10), *F*(1, 263) = 8.37, *p* = .004, *η*^*2*^ = .03.

#### Supplementary analysis

With the same explorative aims as in Study 1, we tested whether the perceived threat mediated the relationship between instability (stability condition coded as 0) and alliance orientation. Results indicated that the mediation was effective (point estimate: 0.23, 95%CI[0.02, 0.46]) reducing the direct effect of instability on alliance orientation to non-significance, *b* = 0.19, *SE* = 0.18, *t*(263) = 1.04, *p* = .299.

### Discussion

Study 2 replicated and extended results from Study 1. Orientation of intermediate-status group members to ally with low-status group was stronger when social stratification was status-detrimental unstable than when it was stable. At the same time, intermediate-status group members supported policies that would presumably help them and other groups to improve social conditions without altering social stratification (i.e., all disadvantaged groups are helped) regardless of stability of social stratification. Thus, instability of social stratification appeared to affect only alliance orientation, suggesting that alliance orientation is a process that is not simply motivated by the desire to achieve social improvement. As in Study 1, this effect was mediated by perceived threat that was induced by instability manipulation. This evidence is congruent with the idea that intermediate-status group members are concerned with maintaining the possibility of positively comparing with the low-status group, and that this concern is stronger when the low-status group is becoming a threat for the social identity of the intermediate-status group.

Unfortunately, a measurement error did not permit taking into account direct help to the low-status group. Thus, we tried to test the effect of stability of social stratification on direct help in Study 3, which also aimed to replicate and generalize results from Study 2 in a different nation.

## Study 3

In Study 3, we test the hypothesis that alliance orientation with a low-status group would increase but direct help to a low-status group and support for helping policies would not when social stratification is status-detrimental unstable. Study 3 was carried out with participants from a different nation (namely Spain) in order to supply evidence of generalizability of the results to other contexts.

### Materials and methods

#### Procedure and participants

Procedure was similar to that used in Study 2, and participants were university students from several humanistic curses at a public university. Procedure was administered in the classroom before lessons by inviting students to complete an online survey about memory and recall of the content of newspapers.

We surveyed 353 voluntary participants. Of them, 61 failed the status manipulation check and were therefore excluded. Ten further participants were excluded because they declared having a different national citizenship. The analyzed sample was thus composed of 282 participants (88.7% female, mean age = 21.58, *SD* = 4.07, range = 18–50). Participants were randomly assigned either to status-detrimental unstable (*n* = 128) or stable (*n* = 154) conditions. Sensitive power analysis indicated that minimum detectable effect size was *η*_p_^*2*^ = .005.

#### Measures

*Alliance with a low-status group orientation*. This was measured with the same three items of Study 2, except that the target nations were different. Answers were rated on a 7-point scale (1 = *completely disagree*; 7 = *completely agree*).

*Support for helping policies and helping an outgroup*. These were measured with the same four items of Study 2, which were rated on a 7-point scale (1 = *completely disagree*, 7 = *completely agree*).

*Interest of ingroup and outgroup and allophilia*. These were measured with the same items as in Study 2 with different target nations [21]. Reliability of allophilia was good (α = .91).

*Manipulation check*. In order to check whether participants were aware of the position of their ingroup, we used the same check item of Study 2. We also added three items asking participants to indicate the status of Germany (high-status outgroup), Spain (intermediate-status ingroup), and Portugal (low-status outgroup) on a 10-point rating scale (1 *= lower status*, 10 = *higher status*). Instability manipulation was checked, as in Study 2, with a 4-item measure of perceived national threat (α = .84) and one item on future differences between Spain and Portugal.

### Results

#### Preliminary analysis

Items measuring alliance orientation, helping the outgroup, and support for helping policies were analyzed with confirmatory factor analysis (maximum likelihood with robust standard error estimation). In this case, the analysis fully supported the expected three-dimension structure, χ^2^(24) = 37.22, *p* = .042, CFI = .98, TLI = .97, RMSEA = .04, *p* = .614, 95% CI [.01, .07], and indicated that all items were adequately measured by the intended latent traits (all *p*s < .001; see S5 Table). Accordingly, reliability was good for alliance orientation (α = .86), helping the outgroup (α = .77, *r*(282) = .63, *p* < .001), and for support for helping policies (α = .76). We then computed the scores of alliance orientation and support for helping policies as latent trait scores. Table 3 depicts the descriptive statistics and zero-order correlations for the considered measures. As indicated, alliance orientation, helping behavior, and support for helping politics were positively correlated. In this study, similarity of national interest correlated with all the dependent measures, supporting the idea that these outcomes are linked to group interests.

**Table 3:** Descriptive statistics and zero-order correlations for the considered measures (Study 3).

	*M*	*SD*	2	3	4	5
**1 Alliance orientation**	.00	1.05	.56**	.42**	.18**	.29**
**2 Helping the outgroup**	.00	0.90	-	.76**	.13*	.45**
**3 Support for helping policies**	.00	0.62		-	.20*	.40**
**4 Similarity of interests**	3.92	1.39			-	.13*
**5 Allophilia**	5.47	1.01				-

*N* = 282.

* *p* < .05.

** *p* < .01.

Again, experimental conditions were equivalent for gender, *χ*^*2*^(1) = 0.14, *p* = .713, mean age, *F*(1, 280) = 0.05, *p* = 0.824, and allophilia, *F*(1, 280) = 0.20, *p* = 0.654.

#### Manipulation check

Replicating the findings of Studies 1 and 2, ANOVA revealed that threat was stronger in the status-detrimental unstable (*M* = 5.12, *SD* = 0.84) than in the stable condition (*M* = 3.45, *SD* = 1.25), *F*(1, 280) = 165.43, *p* < .001, *η*^*2*^ = .37. ANOVA also indicated that, in the status-detrimental unstable condition, participants were more likely to believe that the condition of the ingroup would worsen in the future (*M* = 1.88, *SD* = 0.93) than were participants in the stable condition (*M* = 3.80, *SD* = 1.15), *F*(1, 280) = 232.20, *p* < .001, *η*^*2*^ = .45. Thus, manipulation of stability appeared to be effective.

Regarding concern about the status of the groups, participants rated Germany as higher in status (*M* = 8.20, *SD* = 2.06), Portugal as lower in status (*M* = 3.82, *SD* = 1.69), and Spain (ingroup) as intermediate in status (*M* = 5.22, *SD* = 1.71), *F*(1.42, 398.54) = 773.16, *p* < .001, *η*^*2*^ = .73 (Greenhouse-Geisser correction; all post-hoc *p*s < .001), and this was not affected by the condition of stability either as the main effect, *F*(1, 280) = 1.09, *p* = .296, *η*^*2*^ = .004, or as an interaction, *F*(1.42, 398.54) = 2.15, *p* = .134, *η*^*2*^ = .008.

#### Hypothesis testing

**Fig 2:**
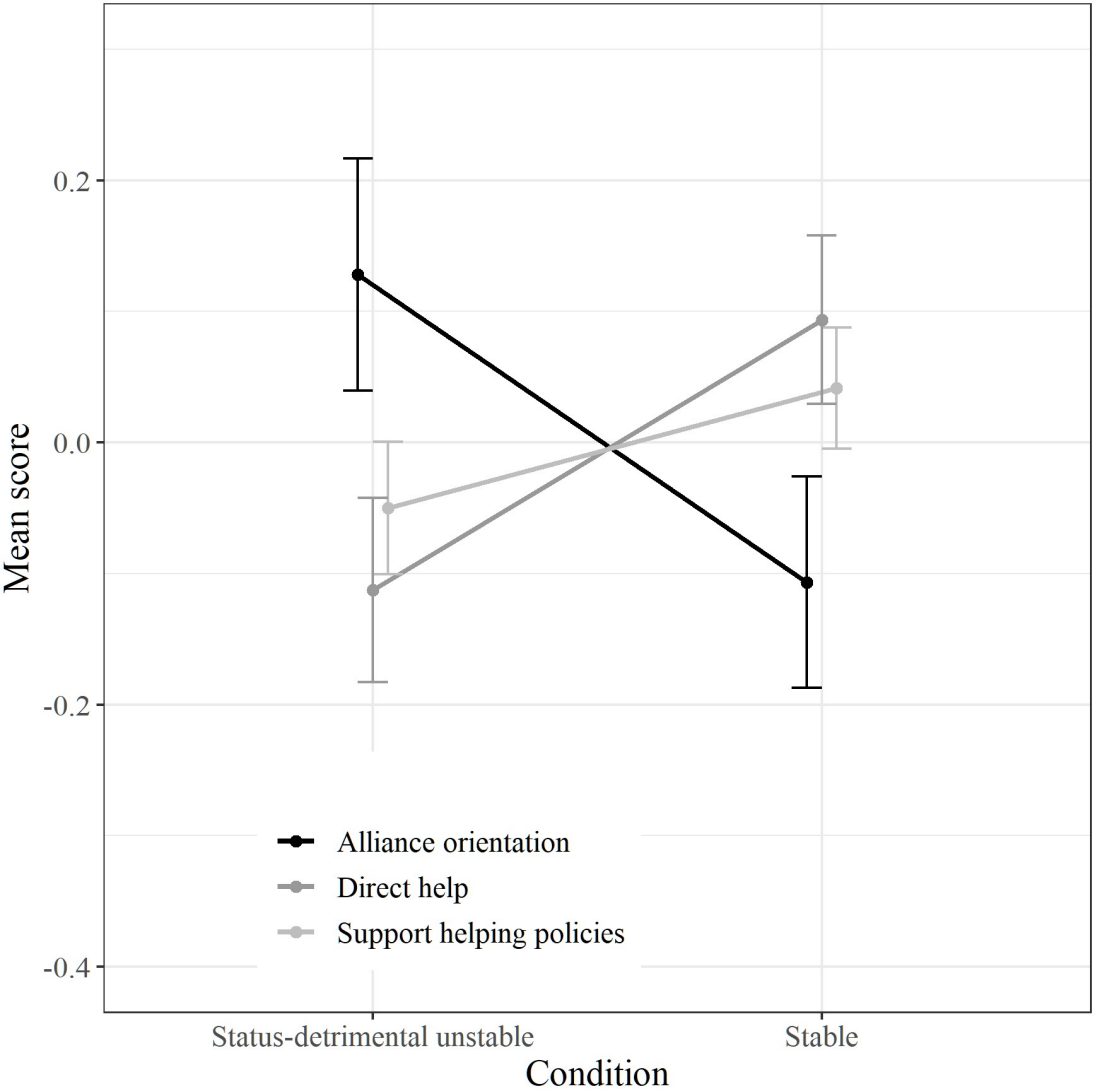
Mean scores of alliance orientation, support for helping policies and direct help according to stability conditions (Study 3). Bars represent standard errors.

A 2 (Stability) x 3 (Alliance vs. Helping outgroup vs. Support for policies) mixed ANOVA was performed with allophilia as covariate (analysis without covariate provided the same results; see S6 Table). In this case, stability had no effect, *F*(1, 279) = .07, *p* = .788, *η*^*2*^ < .001. As expected, a significant interaction appeared, *F*(1.56, 434.32) = 10.74, *p* < .001, *η*^*2*^ = .04 (Greenhouse-Geisser correction). Replicating the findings from Studies 1 and 2, alliance orientation was higher in the status-detrimental instability condition (*M* = 0.13, *SE* = 0.09) than in the stable condition (*M* = -0.11, *SE* = 0.08, *F*(1, 279) = 3.84, *p* = .051), whereas support for helping policies was similar both in the stable (*M* = 0.04, *SE* = 0.05) and in the status-detrimental unstable condition (*M* = -0.05, *SE* = 0.05), *F*(1, 279) = 1.79, *p* = .182. Contrariwise, scores of direct help to the outgroup were higher in the stable (*M* = 0.09, *SE* = 0.06) than in the status-detrimental unstable condition (*M* = -0.11, *SE* = 0.07), *F*(1, 279) = 4.70, *p* = .031. Fig 2 depicts the interaction.

#### Supplementary analysis

Again, mediation analysis revealed that perceived threat mediated the relationship between instability and alliance orientation (point estimate: .29, 95%CI[0.11; 0.49]) and the direct effect of instability turned out to be not significant, *b* = -0.04, *SE* = 0.16, *t*(279) = 0.26, *p* = .795.

### Discussion

Study 3 further supported the findings from Studies 1 and 2 and provided some evidence of their generalizability. First, replicating the results of the first country, intermediate-status group members were more likely to be oriented to ally with a low-status group when social stratification was status-detrimental unstable in the other country as well. Again, perceived threat mediated the relationship between instability and alliance orientation. On the contrary, and similarly to Study 2, support for helping policies favoring less rich nations was not affected by the stability of social stratification. Importantly, direct help to a low-status group did not follow the trend of alliance orientation but, on the contrary, was stronger when social stratification was stable rather than status-detrimental unstable. This supports the hypothesis that alliance with low-status group orientation is strategically aimed at maintaining the social position of the intermediate-status group: when there is a threat to social identity, intermediate-status group members are more oriented to ally with a low-status group but less disposed to directly help that threatening group.

### Combining effects

In order to achieve an overall estimation of the effect of stability on alliance orientation, we meta-analyzed the results of the three studies (we guarantee that those presented in this paper are the only data that we collected to test these hypotheses), transforming the effect size into Cohen’s *d* (high values indicated strong alliance orientation in the status-detrimental unstable condition). Results indicated that, across studies, the overall effect was 0.32 (*SE* = 0.08, *Z* = 4.06, *p* < .001, 95% CI [0.16, 0.47]), with no heterogeneity, *Q*(2) = 0.78, *p* = .68, suggesting that alliance orientation was consistently higher in the status-detrimental unstable condition than in the stable condition (see S2 Fig for the forest plot).

## General discussion

This is the first work to take into account non-competitive intergroup behaviors of intermediate-status groups. Starting from the triadic social stratification theory [2], we showed that intermediate-status groups *may* be oriented to ally with a low-status outgroup when this group is threatening and social stratification is downwardly unstable. This is new evidence and might be regarded as paradoxical, given that, in classic works, a) disadvantaged groups are expected to be more oriented to question the existing social stratification when the social hierarchy is unstable [4,22], and b) a threat from another group is expected to increase prejudice and discrimination against that outgroup [23,24]. The paradox is resolved, however, if one considers that alliance with a threatening outgroup could be strategically aimed at maintaining the existing social stratification. Indeed, seeking alliance with a threatening group might imply that the existing differences between the groups are recognized by all the groups and then maintained, thus preventing the low-status group from further improving its condition. The strategic nature that alliance orientation can acquire is supported by the evidence that intermediate-status group members are more likely to seek alliance with dominant groups, and this is more evident when social stratification is downwardly unstable (Study 1). More importantly, status-detrimental instability does not affect general support for helping policies (Studies 2 and 3) and it decreases the direct help that intermediate-status group members would provide to low-status groups (Study 3). In all studies, moreover, the effect of instability on alliance orientation was mediated by the perceived threat, suggesting thus that alliance orientation was in some way linked to management of the threat. Although the latter evidence should be taken cautiously given that mediation analysis had an explorative purpose, on the whole, these results seem to indicate that alliance orientation among intermediate-status groups might be motivated by the desire to maintain (or not worsen) their intermediary social position (at least when social stratification is downwardly unstable), which allows them to achieve positive intergroup comparison via downward comparison. Through alliance with a low-status group, intermediate-status group members can hope to preserve their social position and, more specifically, the possibility of achieving a positive downward comparison [7,8,19]. In other words, when social stratification is stable, and other things being equal, alliance with a low-status group loses relevance for intermediate-status group members, given that the positive downward comparison is already ensured.

As a matter of fact, one could argue that the present results could also be expected from dominant groups and then observed behaviors are not specific of intermediate-status groups. While we do not disagree with this observation, we would like to highlight that it is the intermediate position of the group to be specific, rather than the behavior. So we believe that if a disadvantaged group behaves as an advantaged one this is not a problem per se but suggests that the classical division between high- and low-status group could be, at least in some cases, not so heuristically sound. This is why the present results also suggest that to adopt an approach that goes beyond a dyadic or binary social stratification can create many opportunities to investigate intergroup coalitions and conflicts. As Simmel [25] suggested more than a century ago, conflict and cooperation are the opposite sides of the same coin, the relationship between dominant and dominated are intrinsically ambivalent, and three elements are oriented to form coalitions of two against one. In Simmel’s view, subordination and superiority are not characteristics of groups but, instead, derive from the existing relations among groups within a hierarchical social organization [4,26]. In such a social game, coalitions and conflicts among groups can change over time and be oriented to different aims depending on the existing social conditions [see, for example, the discussion on the “divide and rule” principle by 10]. Often, then, coalition, alliance, and conflict between groups stem from, and are influenced by, the presence of a third group that is related to other groups.

### Limitations and future directions

One of the principal limitations of this work relies on measurements that were not always as reliable as one would wish. Given the novelty of the present work, we had to develop specific measures, and this is, of course, a point of weakness. However, we tried to refine the measures across studies and to improve procedure from one study to another. For example, it could be argued that we cannot be sure that manipulation of instability in Study 1 was effective given that we used an indirect checking measure (i.e., perceived threat). This is true, but evidence from Studies 2 and 3 give us no reason to believe that the (same) manipulation would not be effective in Study 1. Thus, we believed that the procedure we adopted might increase confidence in the measurements and results. Moreover, the relatively large sample sizes and the use of samples from two different nations as well as the consistency of results across studies seem to indicate that the effects are real and reliable. Another limitation is linked to the use of a generic alliance orientation with other groups. This kind of alliance had no explicit aims (e.g., challenging dominant groups or engaging in social protest) and was simply an orientation to seek some kind of association with other groups. Further, we focused mainly on alliances with low-status groups and did not consider other conditions of instability (e.g., status-ameliorative instability). The focus on these measures and conditions was justified given that the present work aimed to observe the effect of stability of social stratification on alliance orientation among intermediate-status groups, but more research is needed to investigate the process of goal-oriented alliances and coalitions between intermediate-status and other groups.

### Concluding remarks

We are aware that, in this work, we highlighted a “dark-side” of intergroup alliance, that is, the possibility that alliance is aimed at favoring one group over other groups and avoiding social change. However, this is in line with several sociological analyses showing that alliances and conflicts are often two aspects of the same reality [25]. Accordingly, alliance and cooperation are always oriented to achieve some kind of gain, presumably for all parties, but often one part gains more than other parts. To be clear, we are not arguing that this would be an unavoidable outcome and that all instance of alliance are strategically oriented. Rather, in this work, we would suggest that, for a disadvantaged group that can achieve positive social identity by downward comparison, the maintenance of such a possibility might motivate members to seek alliances with a threatening low-status outgroup. This, of course, *does not imply* that intergroup alliances and coalitions are always motivated by the desire to maintain status relations between parties unchanged. Rather, the present results suggest that, for intermediate-status group members, seeking alliance with more disadvantaged groups could be motivated by the desire to manage the threat to social identity that those groups could pose under some circumstances.

## Supporting information

S1 Table Exploratory factor analysis with principal axis factoring and promax rotation on six items about alliance orientation (Study 1). Loadings lower than .30 are omitted.
(DOCX)

S2 Table Results of mixed ANOVA considering alliance orientation scores as the mean of three items (Study 1). (DOCX)

S3 Table Exploratory factor analysis with principal axis factoring and promax rotation on items measuring alliance orientation, direct help to the outgroup, and support for helping policies (Study 2). All = alliance orientation, Hel = Direct help to the outgroup, Sup = Support for helping policies. Loadings lower than .25 are omitted.
(DOCX)

S4 Table Results of stability X alliance vs. support for policies mixed ANOVA without allophilia as covariate (Study 2). Behaviour is the within-participant variable of alliance orientation vs. support for helping policies.
(DOCX)

S5 Table Confirmatory factor analysis with on items measuring alliance orientation, direct help to the outgroup, and support for helping policies (Study 3). *** *p* < .001.
(DOCX)

S6 Table Results of stability X alliance vs. helping outgroup vs. support for policies mixed ANOVA without allophilia as covariate (Study 3). ^a^ Greenhouse-Geisser correction. Behaviour is the within-participant variable of alliance orientation vs. support for helping policies vs. direct help.
(DOCX)

S1 Fig Material used to induce status and manipulate stability of social stratification (Studies 1, 2, 3). (DOCX)

S2 Fig Forest plot of the effect (standardized mean differences) of stability on alliance orientation (Studies 1, 2 and 3). Positive values indicate that alliance orientation was stronger in the status-detrimental unstable condition.
(DOCX)
