# Supplementary material for: Intergroup alliance orientation among intermediate-status group members: The role of stability of social stratification
Source: PLoS One. 2020 Jul 24;15(7):e0235931. doi: 10.1371/journal.pone.0235931 (PMC7380587; doi:10.1371/journal.pone.0235931)
Supplement: S1 Table — Loadings lower than .30 are omitted. (DOCX) [file pone.0235931.s001.docx]

**Table S1.** Exploratory factor analysis with principal axis factoring and promax rotation on six items about alliance orientation (study 1).

| **Item** | ***F1*** | ***F2*** | ***F3*** |
| --- | --- | --- | --- |
| Italy would benefit from an alliance with Germany | .95 |  |  |
| Italy and Germany should make a common front to try to consolidate their position in the European Union | .62 |  |  |
| Italy and Greece should make a common front to try to consolidate their position in the European Union |  | .69 |  |
| Italy would benefit from an alliance with Greece |  | .63 |  |
| Italy should reject any proposal for an alliance with Germany (reversed) |  |  | .73 |
| Italy should reject any proposal for an alliance with Greece (reversed) |  |  | .72 |

Note: Loadings lower than .30 are omitted.
