# Supplementary material for: Intergroup alliance orientation among intermediate-status group members: The role of stability of social stratification
Source: PLoS One. 2020 Jul 24;15(7):e0235931. doi: 10.1371/journal.pone.0235931 (PMC7380587; doi:10.1371/journal.pone.0235931)
Supplement: S2 Table — (DOCX) [file pone.0235931.s002.docx]

**Table S2**. Results of mixed ANOVA considering alliance orientation scores as the mean of three items (Study 1).

| **Effect** | ***df*** | ***F*** | ***p*** | ***η_p_^2^*** |
| --- | --- | --- | --- | --- |
| Stability | 1, 118 | 4.71 | .032 | .04 |
| Direction of alliance | 1, 118 | 18.70 | <.001 | .14 |
| Stability X direction of alliance | 1, 188 | 0.06 | .803 | <.01 |
