# Supplementary material for: Intergroup alliance orientation among intermediate-status group members: The role of stability of social stratification
Source: PLoS One. 2020 Jul 24;15(7):e0235931. doi: 10.1371/journal.pone.0235931 (PMC7380587; doi:10.1371/journal.pone.0235931)
Supplement: S3 Table — All = alliance orientation, Hel = Direct help to the outgroup, Sup = Support for helping policies. Loadings lower than .25 are omitted. (DOCX) [file pone.0235931.s003.docx]

**Table S3**. Exploratory factor analysis with principal axis factoring and promax rotation on items measuring alliance orientation, direct help to the outgroup, and support for helping policies (study 2).

|  | **Item** | ***F1*** | ***F2*** |
| --- | --- | --- | --- |
| All | Italy would benefit from an economic and political alliance with Greece | .92 |  |
| All | Italy would be ‘stronger’ if it would ally economically and politically with Greece | .77 |  |
| All | I am in favor of an economic and political alliance between Italy and Greece | .72 |  |
| Hel | If Greece asked for greater economic aid from Europe, Italy should support Greece | .40 | .38 |
| Sup | I am in favor of a policy that helps the economy of less wealthy European countries to grow |  | .77 |
| Sup | If Europe would decide to economically help the less wealthy European countries, I would agree |  | .69 |
| Hel | I am in favor of a policy to help Greece’s economy to grow |  | .65 |
| Sup | I am in favor of a policy of redistributing the national debt in favor of the less wealthy European nations |  | .50 |
| Sup | The European Union should help the richest nations maintain and increase their well-being rather than helping the less wealthy nations (reversed) |  | -.28 |

Note: All = alliance orientation, Hel = Direct help to the outgroup, Sup = Support for helping policies. Loadings lower than .25 are omitted.
