# Supplementary material for: Intergroup alliance orientation among intermediate-status group members: The role of stability of social stratification
Source: PLoS One. 2020 Jul 24;15(7):e0235931. doi: 10.1371/journal.pone.0235931 (PMC7380587; doi:10.1371/journal.pone.0235931)
Supplement: S4 Table — Behaviour is the within-participant variable of alliance orientation vs. support for helping policies. (DOCX) [file pone.0235931.s004.docx]

**Table S4**. Results of stability X alliance vs. support for policies mixed ANOVA without allophilia as covariate (Study 2).

| **Effect** | ***df*** | ***F*** | ***p*** | ***η_p_^2^*** |
| --- | --- | --- | --- | --- |
| Stability | 1, 264 | 5.06 | .025 | .02 |
| Behaviour | 1, 264 | 0.02 | .897 | < .001 |
| Stability X Behaviour | 1, 264 | 6.11 | .014 | .02 |

Note: Behaviour is the within-participant variable of alliance orientation vs. support for helping policies.
