# Supplementary material for: Intergroup alliance orientation among intermediate-status group members: The role of stability of social stratification
Source: PLoS One. 2020 Jul 24;15(7):e0235931. doi: 10.1371/journal.pone.0235931 (PMC7380587; doi:10.1371/journal.pone.0235931)
Supplement: S5 Table — *** p < .001. (DOCX) [file pone.0235931.s005.docx]

**Table S5**. Confirmatory factor analysis with on items measuring alliance orientation, direct help to the outgroup, and support for helping policies (study 3).

|  |  | Estim. |
| --- | --- | --- |
| Alliance orientation | |  |
|  | I am in favor of an economic and political alliance between Spain and Portugal | .87*** |
|  | Spain would be ‘stronger’ if it would ally economically and politically with Portugal | .79*** |
|  | Spain would benefit from an economic and political alliance with Portugal | .78*** |
| Support for helping policies | |  |
|  | I am in favor of a policy of redistributing the national debt in favor of the less wealthy European nations | .51*** |
|  | If Europe would decide to economically help the less wealthy European countries, I would agree | .83*** |
|  | I am in favor of a policy that helps the economy of less wealthy European countries to grow | .88*** |
|  | The European Union should help the richest nations maintain and increase their well-being rather than helping the less wealthy nations | -.47*** |
| Direct help to the low-status outgroup | |  |
|  | I am in favor of a policy to help Portugal’s economy to grow | .82*** |
|  | If Portugal asked for greater economic aid from Europe, Spain should support Portugal | .77*** |

*** *p* < .001
