# Supplementary material for: Intergroup alliance orientation among intermediate-status group members: The role of stability of social stratification
Source: PLoS One. 2020 Jul 24;15(7):e0235931. doi: 10.1371/journal.pone.0235931 (PMC7380587; doi:10.1371/journal.pone.0235931)
Supplement: S6 Table — a Greenhouse-Geisser correction. Behaviour is the within-participant variable of alliance orientation vs. support for helping policies vs. direct help. (DOCX) [file pone.0235931.s006.docx]

**Table S6**. Results of stability X alliance vs. helping outgroup vs. support for policies mixed ANOVA without allophilia as covariate (Study 3).

| **Effect** | ***df*** | ***F*** | ***p*** | ***η_p_^2^*** |
| --- | --- | --- | --- | --- |
| Stability | 1, 280 | 0.002 | .966 | < .001 |
| Behaviour | 1.59, 445.84^a^ | 0.09 | .875 | < .001 |
| Stability X Behaviour | 1.59, 445.84^a^ | 10.42 | < .001 | .04 |

^a^ Greenhouse-Geisser correction

Note: Behaviour is the within-participant variable of alliance orientation vs. support for helping policies vs. direct help
