# Supplementary material for: Intergroup alliance orientation among intermediate-status group members: The role of stability of social stratification
Source: PLoS One. 2020 Jul 24;15(7):e0235931. doi: 10.1371/journal.pone.0235931 (PMC7380587; doi:10.1371/journal.pone.0235931)

**Figure S1**. Material used to induce status and manipulate stability of social stratification (Studies 1, 2, 3)

**Stable condition**


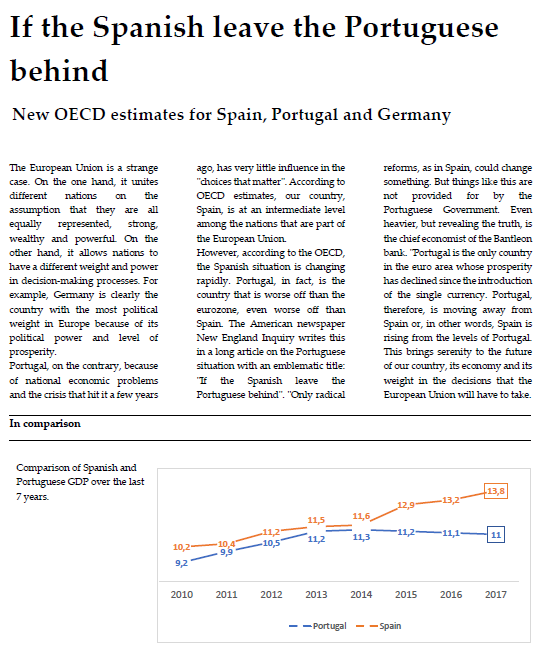


**Status-detrimental unstable condition**


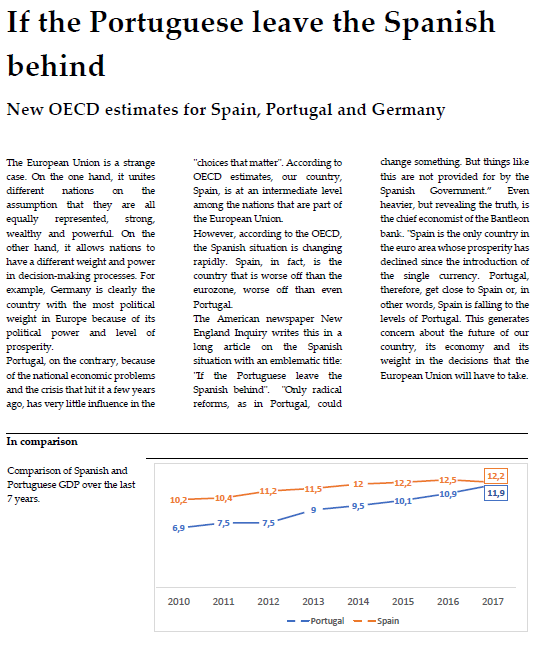

Supplement: S1 Fig — (DOCX) [file pone.0235931.s007.docx]
