# Supplementary material for: Intergroup alliance orientation among intermediate-status group members: The role of stability of social stratification
Source: PLoS One. 2020 Jul 24;15(7):e0235931. doi: 10.1371/journal.pone.0235931 (PMC7380587; doi:10.1371/journal.pone.0235931)

**Figure S2.** Forest plot of the effect (standardized mean differences) of stability on alliance orientation (studies 1, 2 and 3).


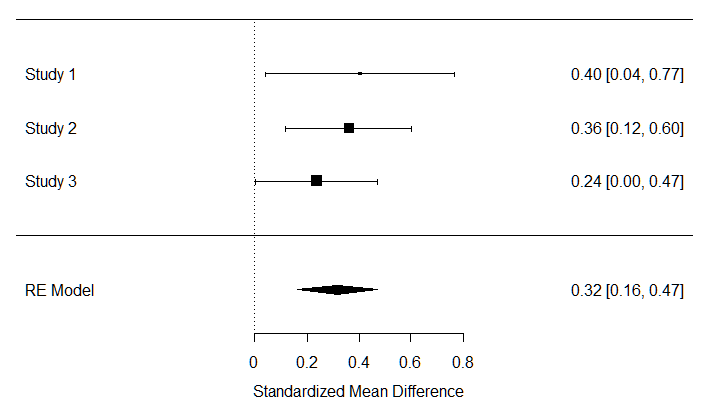

Supplement: S2 Fig — Positive values indicate that alliance orientation was stronger in the status-detrimental unstable condition. (DOCX) [file pone.0235931.s008.docx]
